# Er:YAG Laser-Assisted Root Canal Decontamination in Post-treatement Endodontic Apical Periodontitis: A Case Report

**DOI:** 10.7759/cureus.102709

**Published:** 2026-01-31

**Authors:** Elena Zabrac, Mihaela Chirila, Anca Dragomirescu, Ioana Suciu

**Affiliations:** 1 Department of Endodontics, Carol Davila University of Medicine and Pharmacy, Bucharest, ROU; 2 Department of Orthodontics and Dentofacial Orthopedics, Carol Davila University of Medicine and Pharmacy, Bucharest, ROU

**Keywords:** apical periodontitis, decontamination, laser therapy, lateral canal, retreatment

## Abstract

Bacterial biofilm persistence in the endodontic system is often linked to post-treatment apical periodontitis, which continues to be a significant therapeutic problem. Because of their ability to penetrate and their photothermal effects on resistant microbes, laser technologies have shown great promise in enhancing root canal decontamination. The aim of this brief case report was to assess whether laser decontamination of the root canal system is beneficial for a patient who has persistent apical periodontitis following endodontic treatment. Clinical and radiographic examination of the patient’s teeth revealed post-treatment chronic apical periodontitis at tooth 47, for which endodontic retreatment was indicated. Root canal irrigation was performed using 5.25% sodium hypochlorite, activated with an Er:YAG dental laser to enhance root canal disinfection. Efficacy was assessed using cone beam computed tomography images by comparing the initial and postoperative radiographic evolution. Radiologic assessment at three months confirmed a tendency toward apical lesion regression and clinical improvement, with no postoperative adverse events observed.

## Introduction

Chronic post-treatment apical periodontitis refers to a chronic inflammatory condition of the apical periodontal tissues resulting from failed endodontic treatment. It is usually asymptomatic, and diagnosis is established through radiographic evidence of persistent or enlarging periapical radiolucency [1].

Several factors can facilitate the penetration of bacteria into the endodontic system, most commonly deep carious lesions, coronal-radicular cracks or fractures, attrition, abrasion, and inadequately performed coronal restorations. These conditions compromise the integrity of the dental hard tissues, thereby allowing microbial ingress. Furthermore, microorganisms present in subgingival plaque associated with periodontal disease may invade the pulp tissue through dentinal tubules located in the cervical region of the dental crown [1,2].

Contamination of the root canal system may also occur during or following endodontic treatment procedures. With the removal of the pulp tissue during endodontic treatment, the natural defense mechanisms of the tooth are also eliminated, thereby creating the possibility of secondary contamination of the root canal system. Secondary infection may occur during the course of endodontic treatment, between scheduled appointments, by leakage through temporary restorative material, fracture of the tooth and teeth left open, or after root canal filling, for instance, leakage through temporary or definitive restorative material, recurrent decay, and fracture of tooth structure [3].

Inadequate execution of root canal instrumentation, decontamination, and three-dimensional obturation are major contributing factors to the long-term failure of endodontic treatment. Such deficiencies often lead to the development of chronic apical periodontitis.

Chronic apical periodontitis after endodontic treatment is primarily determined by the persistence of bacteria that survive under adverse conditions in the apical third of the root canal, subsequently inducing inflammation of the periapical tissues. Studies have identified two possible routes of bacterial infiltration following endodontic therapy.

The first involves microbial ingress from uninstrumented or inadequately debrided regions of the root canal that remain after obturation, facilitating the development of secondary intraradicular infection [3]. Microorganisms such as *Enterococcus faecalis* and *Candida albicans*, known for their ability to survive in nutritionally limited environments, have been frequently implicated in the pathogenesis and persistence of post-treatment apical periodontitis.

The second route involves marginal leakage associated with an improper coronal seal [4]. The quality and type of coronal restoration after root canal filling play an important role in the success of endodontic treatment. A well-sealed restoration prevents bacteria and fluids from entering the root canal system, reducing the risk of reinfection. The integrity of the coronal restoration can be evaluated using several criteria: absence of marginal gaps or discrepancies, absence of marginal discoloration, absence of recurrent caries, and no history of restoration decementation. Meeting these conditions helps maintain the integrity of the coronal seal and improves the long-term outcome of the treated tooth [5,6].

Endodontic retreatment is generally considered the preferred approach for failed root canal therapy, especially in cases where the failure is attributed to technical shortcomings during the initial procedure [7].

Root canal retreatment is indicated in situations where traditional endodontic therapy has not achieved a satisfactory outcome. It is often required when clinical or radiographic evidence reveals persistent periapical inflammation or infection, or when postoperative symptoms such as pain, swelling, or sinus tract are present. Furthermore, retreatment is considered appropriate when the tooth is deemed restorable and can be functionally preserved, or when the existing root filling and coronal restoration are inadequate and require replacement to ensure an effective coronal seal and long-term treatment success [8].

Canal decontamination in retreatments follows the same protocols and uses the same solutions as in initial treatments, with ethylenediaminetetraacetic acid at a concentration of 17% and sodium hypochlorite (NaOCl) at 2.5%-5.25% being the most commonly used. Recent studies emphasize the use of activation techniques to enhance the effectiveness of intracanal irrigation, including sonic, ultrasonic, and laser therapy. Endodontic pathologies are primarily caused by bacterial infections, and decontamination strategies that enhance the antimicrobial efficacy of irrigants without inducing adverse effects are considered essential [9,10].

Dental lasers are used in endodontics for two main reasons related to their effects on root dentin: the photothermal effect, which elevates the temperature of the irrigating solution and enhances its antibacterial activity, and the cavitation effect, which clears and opens dentinal tubules, enhancing irrigant diffusion within the canal system [11].

The photoacoustic waves generated during laser activation are highly absorbed by both water and NaOCl, leading to the formation of vapor bubbles that undergo continuous cycles of expansion and collapse upon contact with the canal walls. This phenomenon produces secondary shock waves capable of disrupting the smear layer and unblocking dentinal tubules, thereby facilitating deeper irrigant penetration and improving overall cleaning efficiency [12].

Moreover, noncompliance with manufacturer recommendations, specifically increasing laser output above 3 W, can cause irreversible damage to the root dentin. Proper adjustment of parameters should ensure that the temperature rise within the root canal remains below 5°C to prevent thermal injury [13-15].

## Case presentation

Background

A 38-year-old patient reported mild pain on chewing in tooth 47. Cone beam computed tomography (CBCT) revealed periapical radiolucency involving the mesial and distal roots, and interradicular changes, suggesting failure of previous endodontic treatment.

Clinical examination revealed that tooth 47, without mobility, had no pre-existing mesio-occlusal restoration, and the lingual wall of the crown was fractured. 

Based on these findings, post-treatment apical periodontitis was diagnosed, and the need for endodontic retreatment was determined.

**Figure 1:**
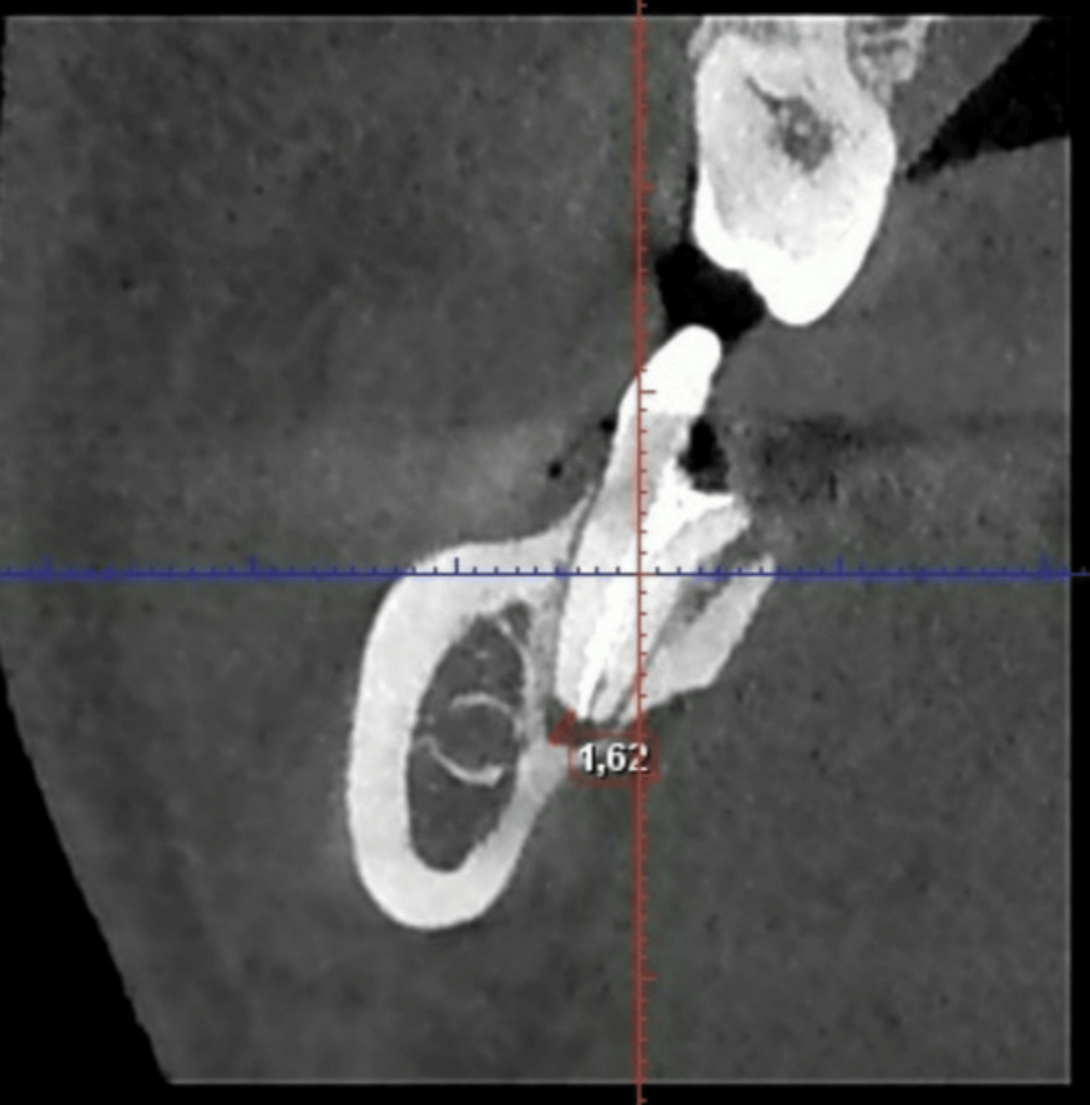
CBCT image of the distal root of tooth 47 showing measurements of the apical defect in coronal section. CBCT, cone beam computed tomography.

**Figure 2:**
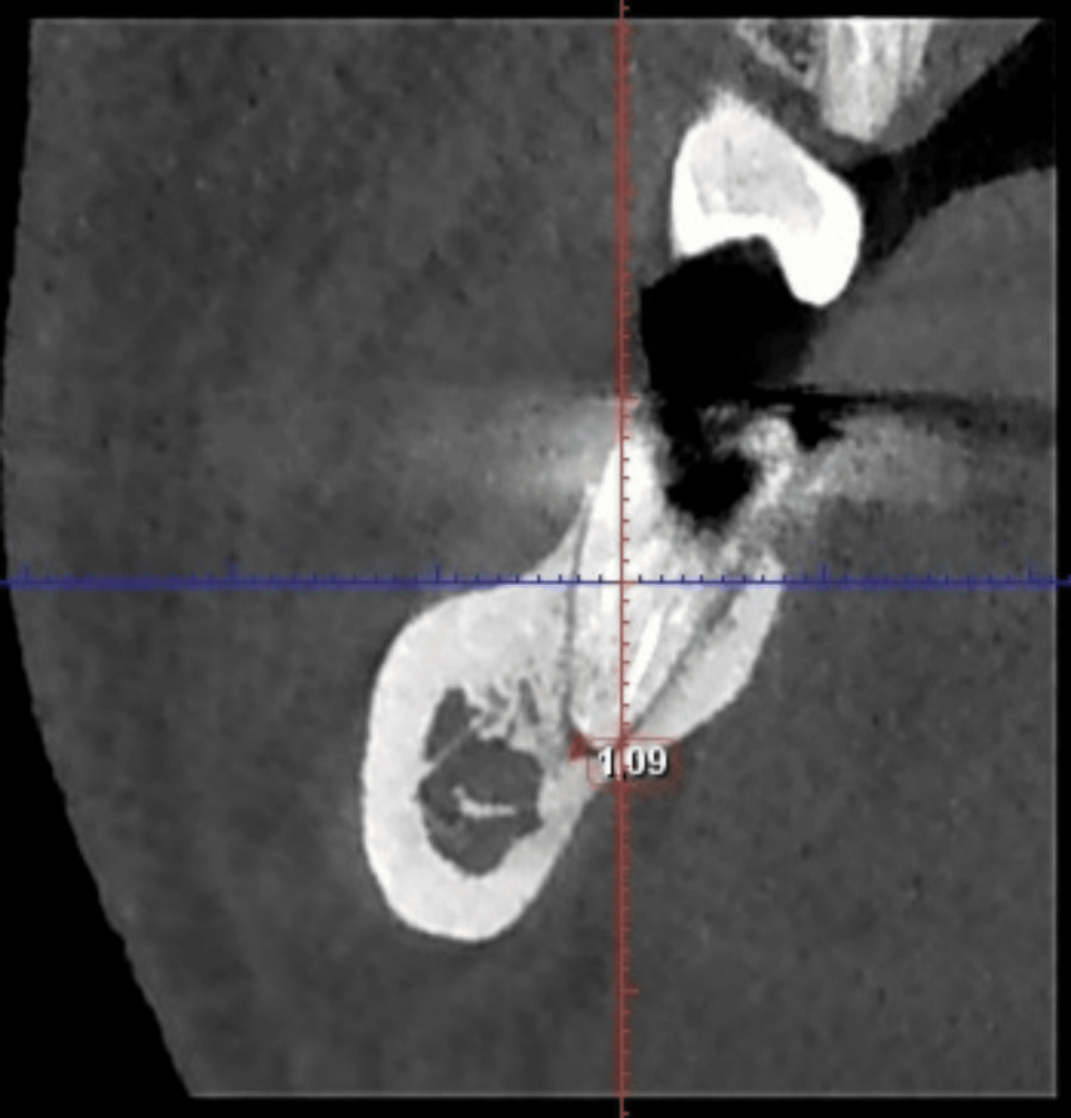
Supplementary CBCT image of the distal root of tooth 47 depicting a different measurement of the apical defect in another coronal section. CBCT, cone beam computed tomography.

**Figure 3:**
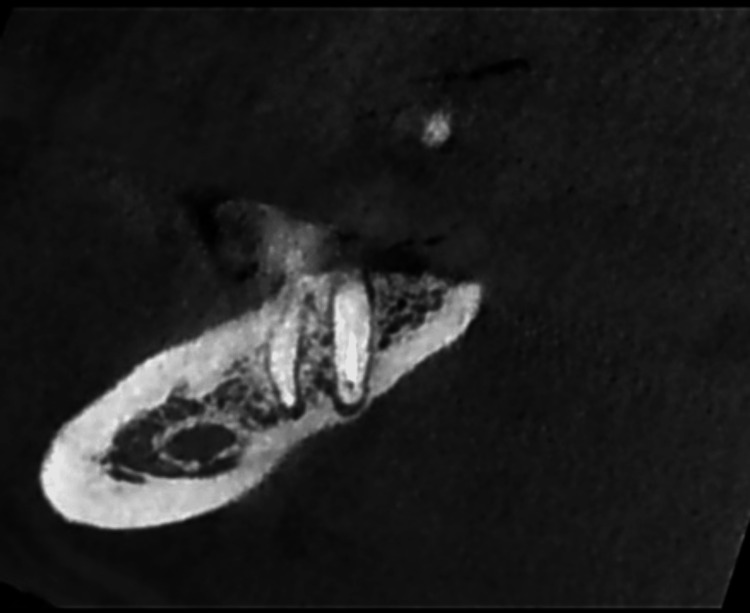
CBCT image of tooth 47 showing the radiolucency on both roots in sagittal section. CBCT, cone beam computed tomography.

CBCT images of tooth 47 (Figures 1-3) are presented to illustrate the presence of periapical lesions following endodontic treatment. Evaluation of the existing root canal filling indicated bacterial leakage and inadequate sealing. 

Figure 2 shows another coronal CBCT section of tooth 47, highlighting the apical aspect of the distal root.

In Figure 3, the sagittal CBCT section reveals radiolucency in the distal and mesial roots.

**Figure 4:**
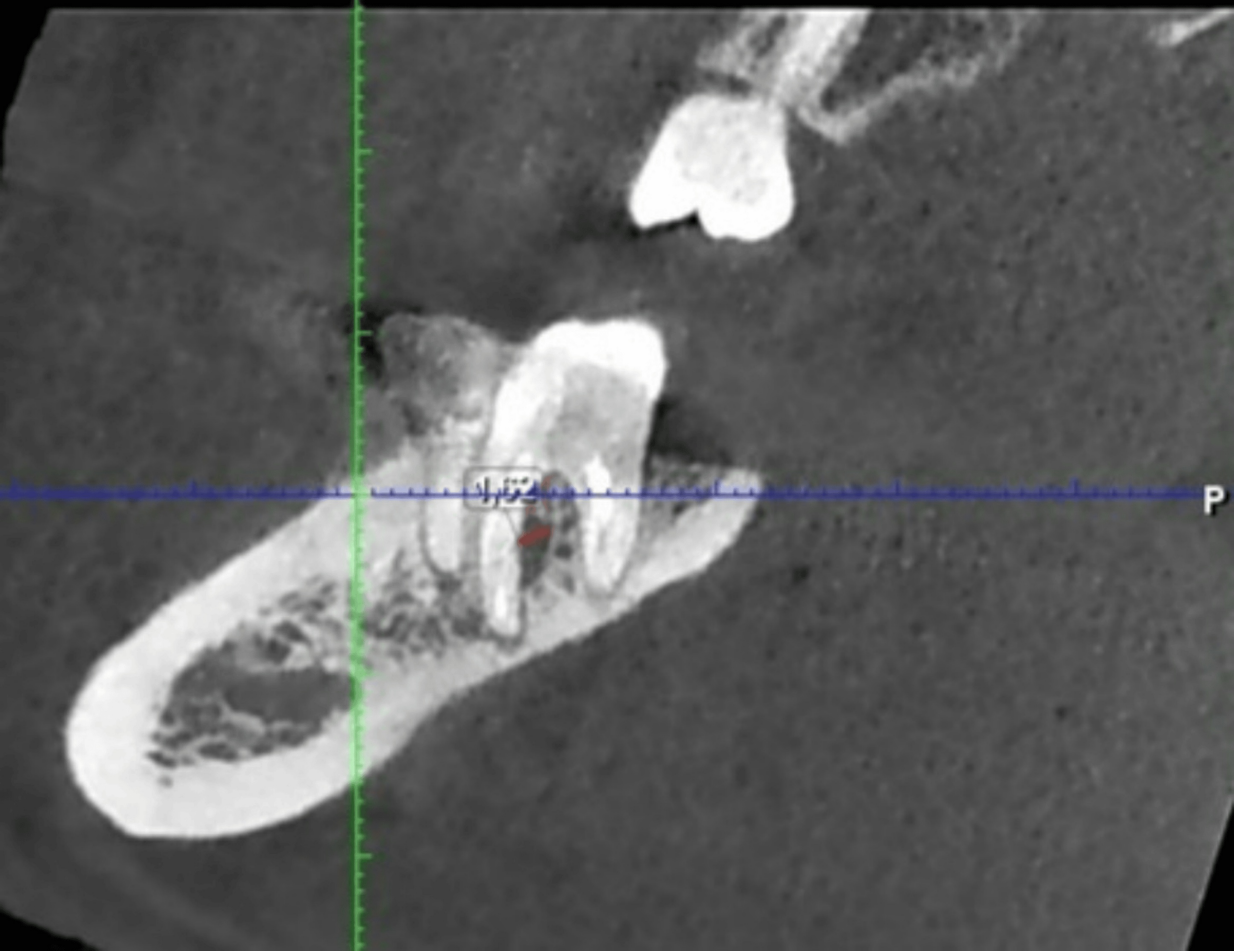
CBCT image of tooth 47 showing measurements of the interradicular defect in sagittal section. CBCT, cone beam computed tomography.

In the following sagittal CBCT section (Figure 4), the interradicular space can be observed, showing the furcal radiolucency.

Treatment procedure

Endodontic retreatment was initiated on all three identified root canals: mesiovestibular (MV), mesiolingual (ML), and distal (D). The existing gutta-percha was removed using the Hyflex Remover (Coltene, Switzerland). Working lengths were established with ISO 10 and 15 Kerr files using the Root ZX mini apex locator (J. Morita, Germany). Each root canal was instrumented to the full working length using the HyFlex EDM single-file rotary system (Coltene, Switzerland) at a speed of 300 rpm and a torque of 2.5 N/cm. For the mesial canals, a #25/.04 rotary file was used, while the distal canal was prepared using a sequential instrumentation protocol with #25/.04 and #40/.04 rotary files.

Root canal irrigation was performed using a 5.25% NaOCl solution, delivered with a 5-mL syringe, with a total volume of 10 mL per canal. Before root canal obturation, the final rinse with 5.25% NaOCl was activated using an Er:YAG dental laser (LightWalker II; Fotona, Ljubljana, Slovenia) in ultra-short pulse mode (25 μs), with a power setting of 0.3 W, a frequency of 15 Hz, and a pulse energy of 20 mJ. The protocol consisted of three laser activation cycles of 30 seconds each per canal, with a resting interval of at least 30 seconds between cycles.

The root canals were dried and prepared for obturation. An apical mineral trioxide aggregate plug was placed in the distal canal due to apical root resorption observed in the CBCT image (Figure 1). The distal canal, as well as the MV and ML canals, were filled using the warm gutta-percha injection technique with Fast-Pack and Fast-Fill system (Eighteeth, China) and AH Plus sealer (Dentsply, USA). A periapical radiograph was obtained to verify the quality of the root canal obturation.

After a two-week period, the patient returned for abutment reconstruction. Therefore, tooth 47 was prepared for a cast metal post. The post space was prepared using Gates-Glidden burs no. 2 and 3, followed by refinement with a Peeso reamer no. 3. The canal preparation was then impressed and sent to the dental laboratory. The fabricated RCR was subsequently cemented with Fuji I glass ionomer cement (GC, Tokyo, Japan).

**Figure 5:**
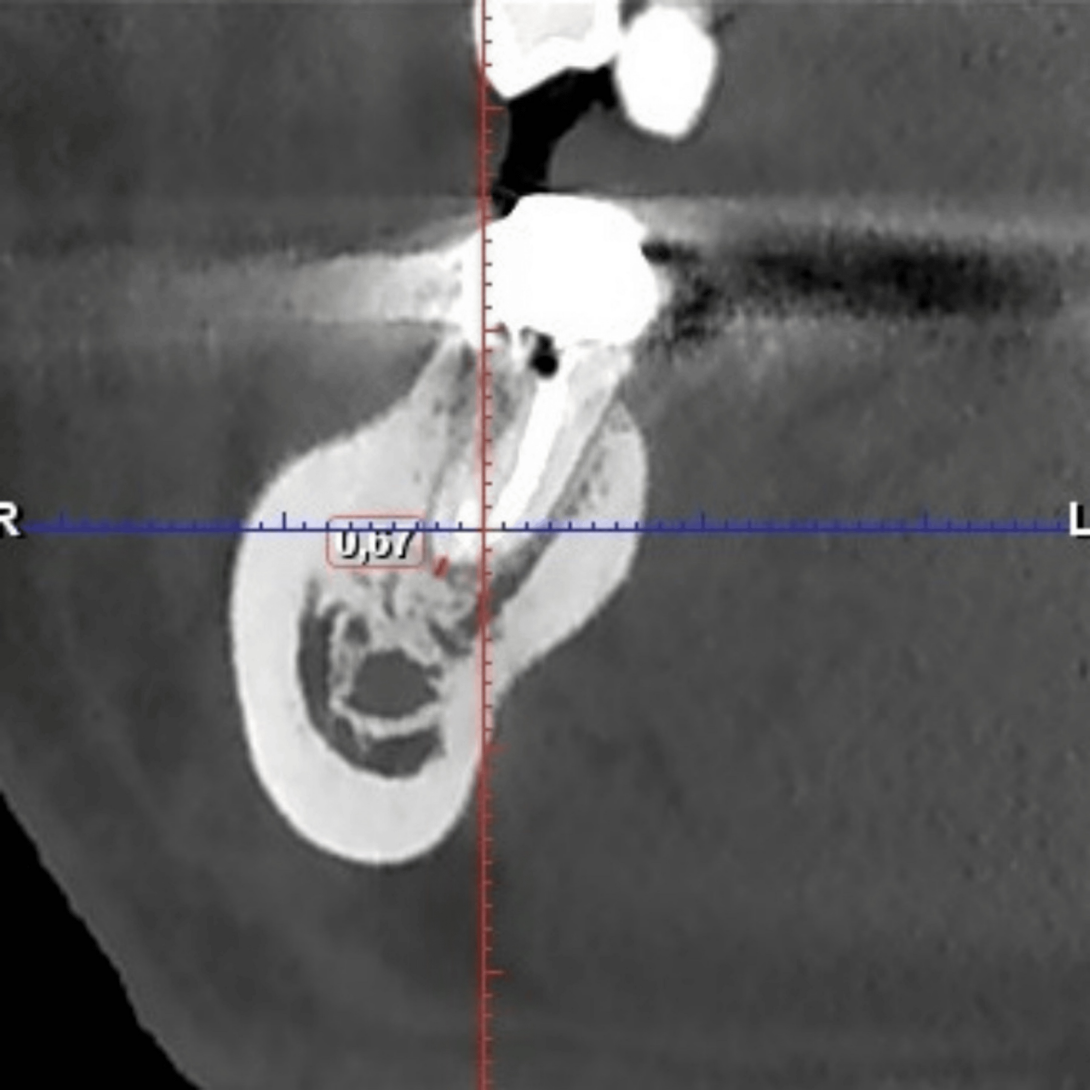
CBCT image of the mesial root of tooth 47 showing measurements of the apical defect, in coronal section, after three months. CBCT, cone beam computed tomography.

**Figure 6:**
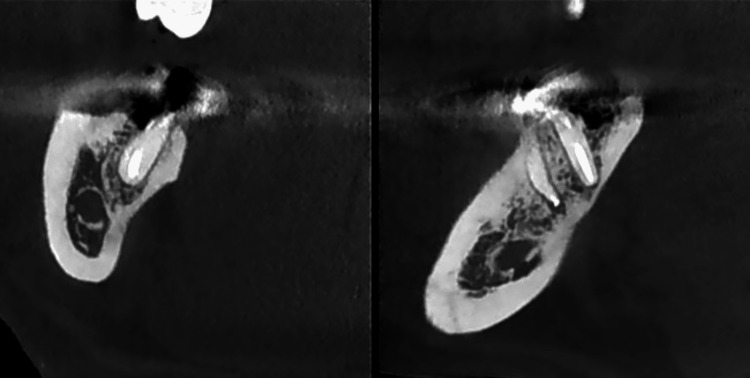
CBCT images of tooth 47, after a three-month period. The left image shows the distal root in the coronal section, while the right image presents the mesial and distal roots in the sagittal section. CBCT, cone beam computed tomography.

**Figure 7:**
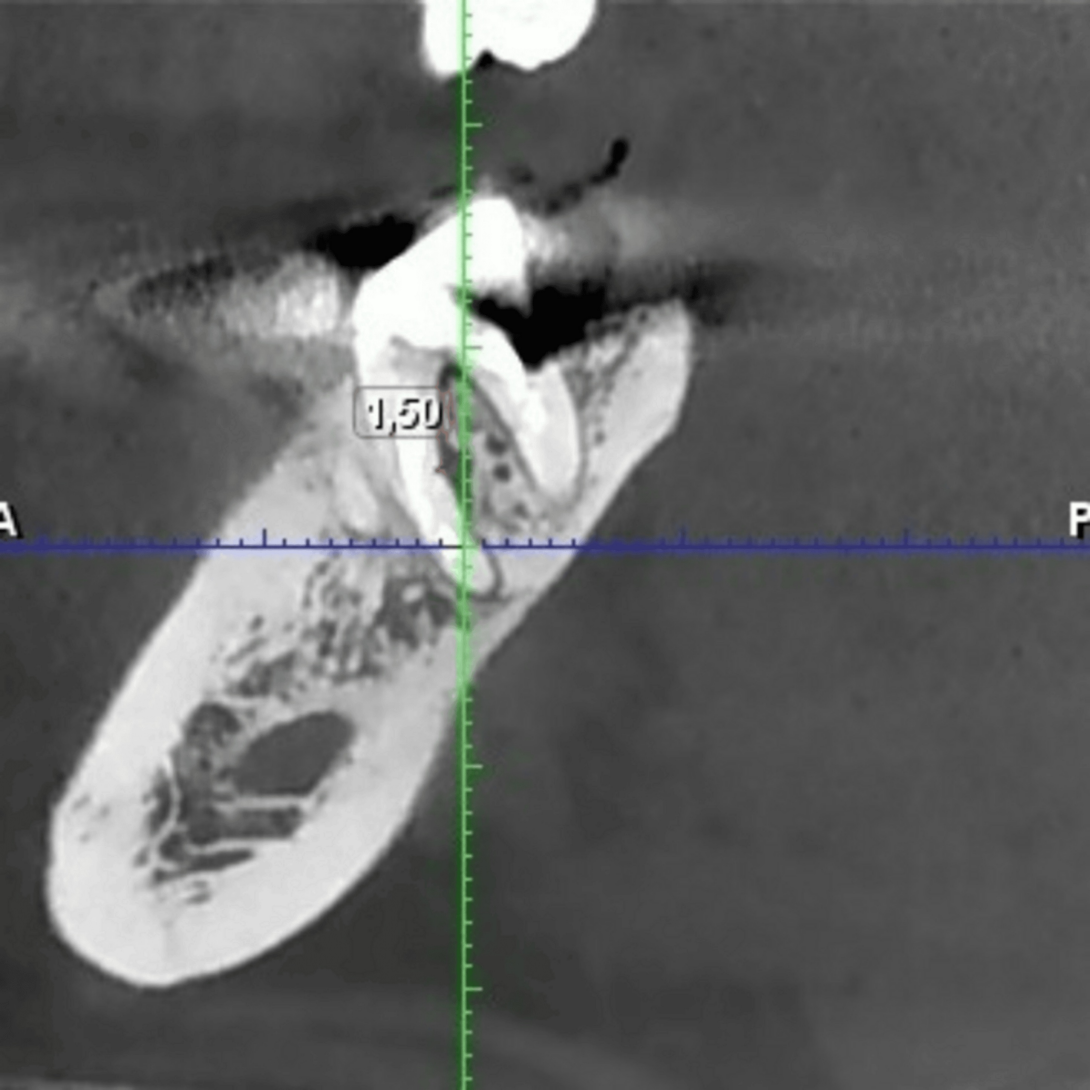
CBCT image of tooth 47 showing measurements of the interradicular defect, in sagittal section, after three months. CBCT, cone beam computed tomography.

At the three-month follow-up of tooth 47, CBCT images (Figures 5-7) show a reduction in apical lesions and a slight improvement in the interradicular area. The tooth remains asymptomatic and is scheduled for crown restoration.

Figure 5 shows a reduction in the size of the radiolucency around the mesial root of tooth 47 following a three-month monitoring period.

Figure 6 demonstrates CBCT images of the distal and mesial roots of tooth 47 after a three-month monitoring period, illustrating the reduction in apical radiolucency.

Figure 7 shows a view of the interradicular space, indicating a tendency toward lesion healing after three months of follow-up.

## Discussion

The CBCT images of tooth 47, shown in Figures 1-3, confirm the diagnosis of post-treatment chronic apical periodontitis. Bacterial infiltration of the root canal filling can be observed, indicated by the lack of homogeneity of the obturation material.

In the coronal section of the distal root (Figure 1), a pronounced periapical radiolucency is evident, corresponding to a bone defect measuring 1.62 mm. In the mesial root (Figure 2), the periapical bone defect measures 1.09 mm.

Laser activation of the final irrigating solution has also been shown to enhance the penetration of sealers into dentinal tubules along the canal walls. However, this technique does not always achieve complete removal of the smear layer or adequate sealer penetration into the apical thirds of curved root canals [14].

The bactericidal effectiveness of Er:YAG and diode lasers depends on both the intensity and duration of laser application. In a 2019 study by Sarda et al., the reduction of *E. faecalis* bacterial cultures following diode laser irradiation of infected canal surfaces was demonstrated [16].

From Figures 4, 7, a reduction in the size of the interradicular defect can be observed, decreasing from 1.62 mm to 1.50 mm in the middle third of the mesial root after a three-month follow-up period. Comparison of interradicular space measurements shows a slight difference of 0.12 mm between pre- and post-retreatment images, indicating that the Er:YAG laser successfully unblocked the lateral canal, allowing irrigation solution penetration.

Given this improvement and the absence of symptoms, the tooth will remain under observation to determine whether periodontal intervention will be necessary.

Research has shown that the use of Er:YAG laser irrigation can lead to less post-treatment discomfort, including milder pain and faster recovery, compared with conventional or ultrasonic irrigation methods [17].

In Figure 5, the coronal section of the mesial root of tooth 47 shows a considerable decrease in apical radiolucency (from 1.09 to 0.67 mm). However, this finding is not confirmed in the sagittal section of tooth 47 (Figure 6), where the contour of the bone resorption remains unchanged compared with the initial examination.

The CBCT image of the distal root of tooth 47 indicates a favorable prognosis, with almost complete resolution of the apical lesion (Figures 1, 6).

Todea et al. demonstrated an improvement in root canal decontamination through the use of the Er:YAG laser, particularly in the coronal and middle segments, resulting in the removal of smear layers and the opening of dentinal tubules. This, in turn, allows for better penetration of the irrigating solution into the lateral canals. Frequently, lateral canals remain contaminated with bacteria or pulp tissue debris following the use of conventional irrigation activation techniques, which may indicate a superior quality of root canal disinfection when the Er:YAG laser is employed [18].

According to Ozbay et al., both laser systems, Er:YAG and Nd:YAG, were not able to ensure complete smear layer removal or full sealer penetration in the apical third of curved root canals [19].

## Conclusions

Although the results are not conclusive, the prognosis appears positive both radiologically and clinically, as indicated by the absence of symptoms. Although longer-term monitoring is required, laser therapy in endodontics has shown effectiveness in unblocking lateral canals. 

Recent advances in root canal disinfection protocols, including the use of laser-activation techniques, aim to enhance the efficiency of smear layer removal from canal walls and hard-to-reach areas, such as the isthmus, lateral canals, and apical delta. However, further clinical studies are required to confirm the effectiveness and reproducibility of the results obtained with dental laser use for root canal disinfection.

## References

[1] Sedani S, Kriplani S, Thakare A, Patel A (2024). The hidden world within: microbial dynamics in root canal systems. Cureus.

[2] Niazi SA, Bakhsh A (2022). Association between endodontic infection, its treatment and systemic health: a narrative review. Medicina (Kaunas).

[3] Siqueira JF, Rôças IN (2022). Treatment of Endodontic Infections.

[4] Siqueira JF Jr, Rôças IN (2008). Clinical implications and microbiology of bacterial persistence after treatment procedures. J Endod.

[5] Hoskinson SE, Ng YL, Hoskinson AE, Moles DR, Gulabivala K (2002). A retrospective comparison of outcome of root canal treatment using two different protocols. Oral Surg Oral Med Oral Pathol Oral Radiol Endod.

[6] Ricucci D, Russo J, Rutberg M, Burleson JA, Spångberg LS (2011). A prospective cohort study of endodontic treatments of 1,369 root canals: results after 5 years. Oral Surg Oral Med Oral Pathol Oral Radiol Endod.

[7] Zanza A, Reda R, Testarelli L (2023). Endodontic orthograde retreatments: challenges and solutions. Clin Cosmet Investig Dent.

[8] Siqueira JF Jr, De Uzeda M, Fonseca ME (1996). A scanning electron microscopic evaluation of in vitro dentinal tubules penetration by selected anaerobic bacteria. J Endod.

[9] Boutsioukis C, Arias-Moliz MT (2022). Present status and future directions - irrigants and irrigation methods. Int Endod J.

[10] Boutsioukis C, Arias-Moliz MT, Chávez de Paz LE (2022). A critical analysis of research methods and experimental models to study irrigants and irrigation systems. Int Endod J.

[11] Suciu I, Zabrac E, Bodnar D, Dimitriu B, Voiculeanu M, Bartok RI, Chirila M (2023). Modern methods of decontamination of the endodontic system. Acta Scientific Medical Sciences.

[12] Tomita Y, Shima A (1986). Mechanisms of impulsive pressure generation and damage pit formation by bubble collapse. J Fluid Mech.

[13] Miserendino LJ, Abt E, Wigdor H, Miserendino CA (1993). Evaluation of thermal cooling mechanisms for laser application to teeth. Lasers Surg Med.

[14] Gupta R, Wadhwani KK, Tikku AP, Chandra A (2020). Effect of laser-activated irrigation on smear layer removal and sealer penetration: an in vitro study. J Conserv Dent.

[15] Zabrac E, Suciu I, Dimitriu B, Ciocîrdel M, Nistor CC, Bartok R, Amza O (2025). A microscopic study analysing the extension of a burn lesion produced „în vitro” on radicular dentin using the dental laser. Rom J Oral Rehabil.

[16] Sarda RA, Shetty RM, Tamrakar A, Shetty SY (2019). Antimicrobial efficacy of photodynamic therapy, diode laser, and sodium hypochlorite and their combinations on endodontic pathogens. Photodiagnosis Photodyn Ther.

[17] Mandras N, Pasqualini D, Roana J (2020). Influence of photon-induced photoacoustic streaming (PIPS) on root canal disinfection and post-operative pain: a randomized clinical trial. J Clin Med.

[18] Todea DCM, Luca RE, Bălăbuc CA, Miron MI, Locovei C, Mocuta DE (2018). Scanning electron microscopy evaluation of the root canal morphology after Er:YAG laser irradiation. Rom J Morphol Embryol.

[19] Ozbay Y, Erdemir A (2018). Effect of several laser systems on removal of smear layer with a variety of irrigation solutions. Microsc Res Tech.

